# Human papillomavirus infection and vaccination with thyroid diseases: mechanistic links and immunological insights

**DOI:** 10.3389/fimmu.2025.1732358

**Published:** 2025-12-17

**Authors:** Zhengda Wang, Xiwen Chang, Xianbin Cheng

**Affiliations:** 1 The Orthopaedic Medical Center, The Second Hospital of Jilin University, Changchun, Jilin, China; 2 Joint International Research Laboratory of Ageing Active Strategy and Bionic Health in Northeast Asia of Ministry of Education, Changchun, Jilin, China; 3 Department of Breast Surgery, The Second Hospital of Jilin University, Changchun, Jilin, China; 4 Department of Thyroid Surgery, The Second Hospital of Jilin University, Changchun, Jilin, China

**Keywords:** human papillomavirus, immunological insights, mechanistic, thyroid diseases, vaccination

## Abstract

Human papillomavirus (HPV) is one of the most prevalent DNA viruses worldwide. Beyond its well-established oncogenic roles in cervical and head and neck squamous cell carcinomas, accumulating evidence suggests that HPV may also contribute to the pathogenesis and progression of thyroid diseases. This review systematically integrates recent advances from histological, molecular, and epidemiological studies to comprehensively evaluate the potential associations between HPV infection and different types of thyroid disorders. The findings indicate that HPV infection may promote thyroid dysfunction and tumor development by inducing chronic inflammation and disrupting immune homeostasis, whereas HPV vaccination appears to exert a protective effect by restoring immune homeostasis. This study establishes an integrative framework linking HPV infection, immune modulation, and thyroid diseases, providing new insights into the pathophysiological mechanisms of virus-related thyroid disorders and a theoretical foundation for future immunotherapy and precision prevention research.

## 1 Introduction

Human papillomavirus(HPV) is among the most common viruses worldwide, with more than 200 genotypes identified to date. High-risk types, particularly HPV16 and HPV18, are responsible for approximately 70% of cervical cancer cases (1, 2). According to the World Health Organization(WHO), the global prevalence of HPV infection among women is about 11–12%, and it exceeds 20% in certain Asian countries (3, 4). As a preventive vaccine against viral oncogenesis, the HPV vaccine has substantially reduced high-risk HPV infections and related precancerous lesions worldwide. Beyond this, it is increasingly recognized for its broader capacity to modulate host immune homeostasis (5–7). With widespread vaccination, interest has grown in its potential systemic immunoregulatory effects, as several studies suggest that HPV vaccines may offer not only local protection but also systemic immune benefits in other immune-sensitive organs (8, 9). The thyroid gland, one of the most immunologically active endocrine organs, is particularly susceptible to viral infections and inflammatory responses (10). Its exceptionally rich vascularization and heightened immune responsiveness further increase its sensitivity to viral antigens, inflammatory mediators, and systemic immune perturbations (11, 12). Previous studies have reported associations between viral infections such as Epstein–Barr virus(EBV), parvovirus B19, and herpes simplex virus, and the development of thyroiditis or papillary thyroid carcinoma(PTC) (13–16). Within this context, HPV, as a virus capable of inducing chronic inflammation and immune evasion, has recently been proposed to participate in thyroid pathology.

Histological and molecular studies have detected HPV DNA or p16 positivity in PTC and primary squamous cell carcinoma, suggesting limited viral presence in thyroid tissue (17, 18). Epidemiological data further show that prior HPV infection increases thyroid cancer risk, whereas HPV vaccination is associated with a lower incidence of thyroid diseases (19). A clearer understanding is limited by the lack of studies that bring together evidence from different angles, despite the rapidly expanding amount of available data. Most existing investigations focus on only one dimension, making it difficult to capture broader patterns at the population level. In light of this, the present review aims to systematically integrate existing molecular, immune, and population-based evidence. This approach allows a comprehensive exploration of the potential roles and interrelationships between HPV infection and HPV vaccination in thyroid diseases. To delineate the scope of this analysis, the discussion focuses on autoimmune thyroid disorders and neoplastic conditions, particularly papillary thyroid carcinoma, where HPV-related mechanisms have been most frequently proposed. By integrating evidence from multiple dimensions, this review outlines a coherent framework to interpret how HPV-related immune disturbances might influence thyroid pathology. This perspective offers useful direction for future immunotherapeutic and precision-prevention strategies, especially in individuals with both viral exposure and endocrine susceptibility.

## 2 HPV infection to thyroid diseases

### 2.1 Histological and molecular evidence

Histopathological evidence suggests that HPV may play a potential role in certain thyroid tumors. Several studies on PTC have reported the presence of viral DNA in tumor tissues, indicating a possible tissue-specific distribution of HPV within the thyroid gland. In a study including 82 PTC cases and 77 benign nodules, HPV DNA was detected in 13.4% and 3.8% of samples, respectively, whereas all adjacent normal tissues tested negative (P<0.05). Multivariate analysis further confirmed a significant association between HPV infection and PTC (OR = 4.77, 95%CI: 1.17–19.40) (20). Moreover, patients with HPV-positive PTC exhibited a higher incidence of capsular invasion, suggesting that viral infection may contribute to local tumor aggressiveness. Another investigation analyzing 157 thyroid tumor samples also reported the presence of HPV DNA, with an overall positivity rate of 2.5%. Specifically, HPV-33 was identified in two PTC samples, while HPV-6 was found in two hyperplastic thyroid lesions. Most positive cases occurred in female patients, and the detection of high-risk HPV-33 supported its potential association with abnormal epithelial proliferation in the thyroid gland (21). Ramadan and colleagues further substantiated the link between HPV infection and thyroid tumors. In a cohort of 70 thyroid tissue specimens, HPV DNA was detected in both malignant and benign lesions, while all normal thyroid samples were negative (P<0.05). High-risk HPV-16 and HPV-18 were predominantly found in malignant tissues, whereas low-risk HPV-6 and HPV-11 were also occasionally detected. Notably, HPV positivity was significantly correlated with obesity, suggesting that a metabolic inflammatory environment may facilitate viral oncogenesis (22). Cao et al. reported a rare case involving a patient with HPV-associated base-of-tongue squamous cell carcinoma (SCC) and small cell neuroendocrine carcinoma (SCNEC). The patient simultaneously presented with metastatic papillary thyroid carcinoma in the cervical lymph nodes. Both SCC and SCNEC components were strongly positive for p16 but negative for EBER, indicating HPV-driven tumorigenesis. This coexistence of lesions within a single patient suggests that HPV may not only replicate in epithelial tissues but also influence the thyroid microenvironment through local immune or inflammatory pathways (23). Similarly, Hayashi et al. described a case in which a patient developed thyroid metastasis five years after surgery for cervical SCC. The metastatic thyroid lesion exhibited strong p16 positivity and morphological features identical to the primary cervical carcinoma, while TTF-1 negativity confirmed its origin as metastatic HPV-related cervical cancer. A summary of 14 reported cases of cervical cancer metastasizing to the thyroid showed that most primary tumors were HPV-positive squamous carcinomas with long latency periods. These findings imply that HPV-related cancer cells may retain the ability to survive and proliferate within thyroid tissue (24). Additionally, Head et al. documented a patient with HPV-positive oropharyngeal carcinoma who was found to have a concomitant occult papillary thyroid carcinoma. In this case, the oropharyngeal tumor was positive for both E6/E7 mRNA and p16, while the thyroid lesion was HPV-negative but displayed marked local inflammatory infiltration. This observation raises the possibility that HPV infection may promote thyroid tumorigenesis indirectly through paracrine signaling or microenvironmental remodeling (25). Taken together, these findings underscore the multifaceted role of HPV within thyroid tissue. The virus may contribute directly to tumor initiation through viral gene integration or act indirectly by modulating immune and inflammatory pathways that alter the tumor microenvironment.

At the molecular level, rare cases of primary squamous cell carcinoma of the thyroid (PSCCT) have provided important clues supporting HPV involvement in thyroid tumorigenesis. Recent case analyses have demonstrated diffuse p16 positivity in both PSCCT and coexisting PTC regions, with molecular assays confirming the presence of HPV-6 or HPV-33 DNA. In addition, BRAF V600E mutations were detected in the PTC components (26, 27). The coexistence of HPV infection and BRAF mutations suggests that the virus may promote cell-cycle dysregulation through activation of the p16/CDK4–Rb pathway. This interaction may cooperate with oncogenic driver mutations to enhance tumor transformation and increase intratumoral heterogeneity. This finding provides molecular evidence of a potential synergistic mechanism between HPV infection and thyroid malignant transdifferentiation, broadening the pathological spectrum in which viral and genetic oncogenic events coexist. Although several studies failed to detect HPV DNA in benign thyroid lesions, these negative results reinforce the notion that the virus may exhibit preferential distribution in malignant tissues. In contrast, EBV showed higher detection rates in the same sample sets, indicating that distinct viruses may participate in thyroid microenvironmental regulation through different immunological patterns (28, 29). Molecular analyses of malignant thyroid tumors have also identified HPV DNA in approximately 13.4% of PTC tissues. This prevalence is significantly higher than that observed in benign nodules and adjacent normal thyroid tissues. The detection of high-risk HPV-33 and low-risk HPV-6 further suggests that viral genotypes may influence oncogenic potential. A recent study systematically screened benign thyroid disorders, including Hashimoto’s thyroiditis, adenomas, and cases with both conditions, for viral infections. HPV DNA was not detected in any of the samples, whereas EBV DNA was present in 55.2%, 37.6%, and 67.6% of the respective groups. These findings suggest that HPV is unlikely to be a common infectious agent in benign thyroid diseases, while EBV may play a more prominent role in shaping the local immune environment (30). Another study investigated the presence of herpesviruses and polyomaviruses in thyroid carcinoma tissues, including EBV, HSV, CMV, BKV, and SV40. Although HPV DNA was not detected, EBV and polyomavirus sequences were identified in several thyroid cancer specimens, suggesting that viral infections may exert complex comorbid effects in the pathogenesis and progression of thyroid malignancies (31, 32). Taken together, while HPV infection is not a ubiquitous event in thyroid tissue, this variability may reflect differences in detection sensitivity, tissue preservation, and geographic factors. It is also possible that study-specific factors such as sample type, PCR primer selection, and genotyping panels contribute to the wide range of reported positivity rates, rather than indicating true biological absence or presence of the virus (33, 34). Nevertheless, the detection of HPV DNA in selected PTC and PSCCT cases supports the hypothesis that HPV may act as a co-carcinogenic factor. Its coexistence with oncogenic signaling mutations further suggests a potential contribution to thyroid cancer initiation and progression. It is worth noting that HPV genotypes differ markedly in their biological relevance (35–37). High-risk types such as HPV16, HPV18, and HPV33 are more prone to integrate into the host genome and exert stronger E6/E7-mediated effects on cell-cycle and immune regulation. In contrast, low-risk types such as HPV6 and HPV11 are found mainly in benign or hyperplastic thyroid lesions, suggesting a genotype-dependent gradient in endocrine tropism and oncogenic potential. Overall, these studies collectively indicate that HPV DNA can be detected in both benign and malignant thyroid tissues, although the prevalence remains low and variable across studies. Future studies employing high-sensitivity molecular assays and large multicenter cohorts are warranted to clarify the true oncogenic role of HPV in thyroid neoplasia.

### 2.2 Clinical and epidemiological evidence

In recent years, a growing body of clinical and epidemiological evidence has suggested a significant association between HPV infection and the development of thyroid cancer. A large-scale case–control study by Yang et al. reported that the prevalence of prior HPV infection was markedly higher in patients with thyroid cancer than in controls (15.3% vs. 7.6%, P<0.001). Multivariate regression analysis confirmed that HPV infection was independently associated with thyroid cancer (OR = 2.199, 95%CI: 1.939–2.492), with no significant sex-related differences (male OR = 2.48; female OR = 2.12) (38). As a case–control study, these findings offer reliable comparative evidence, although this design can only indicate association rather than establish temporal causality. These findings indicate a significant epidemiological association between HPV infection and thyroid cancer, although some of the available data do not yet support a direct causal relationship. Mechanistically, high-risk HPV types are hypothesized to influence thyroid tumorigenesis, in part through E6- and E7-associated effects on p53 and Rb pathways, although these interactions remain insufficiently characterized in thyroid-specific models (39, 40). With widespread implementation of HPV vaccination, the incidence of thyroid cancer may potentially decrease in the future. Meng et al. conducted a meta-analysis including 11 clinical studies comprising 1,432 patients with thyroid cancer. The pooled results showed that HPV infection significantly increased thyroid cancer risk (OR = 2.20, 95%CI: 1.31–3.23, P<0.05), with minimal heterogeneity (I²=0%). Because meta-analyses synthesize results from multiple independent datasets, the conclusions drawn from this analysis carry higher evidentiary weight and reduce the influence of study-level variability. The authors proposed that high-risk HPV types, especially HPV-16 and HPV-18, can integrate into the host genome, leading to p53 and Rb pathway inactivation through the actions of the E6 and E7 proteins. This process promotes cell-cycle dysregulation and genomic instability, which in turn drive thyroid carcinogenesis. Moreover, HPV positivity was found to be more frequent in Asian populations, suggesting possible geographic and ethnic variations (41). From a broader perspective, Mostafaei et al. examined the overall association between viral infections and thyroid cancer in a systematic review of 23 studies. Their analysis revealed that viral infections collectively increased thyroid cancer risk, with HPV, EBV, and parvovirus B19 identified as the principal high-risk viruses. HPV DNA sequences have been detected in several thyroid tumor tissues, particularly in papillary and follicular subtypes, supporting the hypothesis that viral infection may contribute to tumorigenesis through chronic inflammation, oxidative stress, and immune evasion mechanisms (42). A radiological study from Europe provided additional clinical insights by comparing imaging characteristics of HPV-associated lesions with those of thyroid cancer. Among 90 patients with cystic cervical lesions, HPV-positive metastatic oropharyngeal carcinoma frequently exhibited cystic transformation patterns closely resembling the ultrasonographic features of PTC (43). Li et al. further reported that HPV infection may influence thyroid function and cancer susceptibility. HPV DNA was detected in 13.4% of PTC tissues compared with 3.8% in benign nodules, a difference that reached statistical significance. The authors noted that this association appeared to be stronger in female patients (44). In addition, large-scale population data have demonstrated an inverse relationship between HPV vaccination and thyroid disease risk, suggesting a potential protective effect of the vaccine. Taken together, converging evidence from clinical observations, case–control studies, meta-analyses, and molecular investigations consistently supports a close relationship between HPV infection and increased thyroid cancer risk, particularly in PTC. HPV may represent a modifiable oncogenic risk factor, offering new perspectives for early screening, prevention, and targeted intervention in thyroid malignancies(**Table 1**).

**Table 1:** Main findings of studies on HPV infection and thyroid diseases.

Study design	HPV evidence	Thyroid disease	Key findings	Reference
Cross-sectional	HPV DNA detected by PCR: 13.4% in PTC, 3.8% in benign nodules	PTC	HPV positivity significantly higher in PTC than benign nodules and normal tissue	(20)
Cross-sectional	PCR-RFLP for HPV 6 and 33	PTC, FTC and ATC	HPV-6/33 found in hyperplasia and PTC, suggesting possible viral involvement in thyroid neoplasms.	(21)
Case–control	Real-time PCR for HPV 6, 11, 16, 18, 33, 58	PTC and FTC	HPV DNA detected in 44.4% of cancers vs 36.0% of benign and 0% of normal tissues	(22)
Case–control	HPV-6 detected in 2 cases, HPV-33 in 1 case	PSCCT and PTC	HPV DNA identified in both PSCCT and PTC	(26)
Postoperative tissue	Real-time PCR for HPV16/18, 1/40 positive	PTC	HPV16/18 DNA detected in PTC	(28)
Cross-sectional	15.3% in TC vs. 7.6% in controls	Thyroid cancer	HPV infection significantly associated with higher thyroid cancer risk	(38)
Case–control	HPV-18 L1 IgG measured by ELISA	Thyroid eye disease	HPV18 L1 antibody levels significantly increased in chronic and active TED	(45)
Transgenic mouse models	Thyroglobulin promoter–driven expression of HPV16 E7	Thyroid lesions and PTC	RET/PTC3 mice showed 28% tumour rate; E7 phenotype dominant in co-expression.	(46)

### 2.3 Immunological mechanisms of HPV-induced thyroid pathogenesis

Understanding the immunological basis of HPV infection helps clarify whether the epidemiological patterns observed reflect causal processes or secondary immune effects, and explains how viral persistence and immune evasion could influence thyroid biology. Beyond its well-established oncogenic roles in cervical and head and neck squamous cell carcinomas, HPV is also involved in the initiation and progression of thyroid diseases through multiple immunological pathways. Recent evidence outlines several biologically plausible routes through which HPV-related signals may reach or influence thyroid tissue. HPV DNA has been identified in circulating peripheral blood mononuclear cells, suggesting that viral material may enter the bloodstream and disseminate beyond epithelial sites (34). Rare reports of HPV-positive cervical carcinoma metastasizing to the thyroid, with metastatic lesions retaining p16 positivity, further indicate that HPV-driven cells are able to survive within the thyroid microenvironment, which implies potential transport through lymphatic or hematogenous pathways (47). Viromics studies also show that HPV-derived nucleic acids can be packaged into plasma exosomes and transported systemically (48). These mechanisms outline how HPV-related signals may reach the thyroid, providing a basis for understanding their subsequent immunological effects. Current evidence suggests that HPV does not primarily act by directly infecting thyroid cells. Instead, it influences thyroid pathophysiology indirectly through mechanisms such as immune evasion, chronic inflammation, molecular mimicry, oxidative stress, and co-infection with other viruses. This persistent remodeling of the immune microenvironment exerts dual effects. On one hand, prolonged immune activation and inflammatory stress can drive structural remodeling and functional imbalance within thyroid epithelial tissues. On the other hand, sustained immune dysregulation may trigger or aggravate autoimmune responses against thyroid antigens, leading to a pathological state characterized by both chronic inflammation and immune tolerance.

At the molecular level, the E6 and E7 oncoproteins of HPV are central mediators of immune regulation (43, 49). The roles of E6 and E7 in immune modulation have been demonstrated in thyroid samples as well as in cervical and oropharyngeal cancers, suggesting a conserved set of viral strategies with tissue-specific variations. E6 suppresses p53-dependent pathways, thereby reducing apoptosis and antiviral responses, whereas E7 binds to and inactivates the retinoblastoma protein(Rb), releasing E2F transcription factors and sustaining continuous cell-cycle activation and DNA replication (1). Persistent cell-cycle dysregulation weakens host immune surveillance and downregulates antigen-presenting molecules such as MHC-I, leaving cells in a state of reduced apoptosis and heightened immune stress (50). Overexpression of p16, often regarded as a molecular marker of persistent HPV infection, reflects this imbalance (51). During this stage, cells exhibit enhanced proliferative signaling, impaired antiviral defense, and reduced immune recognition, providing an immunological foundation for subsequent chronic inflammation. Within this context, HPV infection can further induce inflammatory cascades and activate multiple immune-related signaling pathways (42). E6 and E7 proteins have been shown to continuously activate the NF-κB, STAT3, and PI3K/Akt pathways, leading to upregulation and sustained secretion of cytokines such as IL-6, TNF-α, IL-1β, and TGF-β (52, 53). These signaling alterations are well established in other HPV-driven tumors and are increasingly being explored in thyroid disease contexts. This amplification of signaling promotes immune-cell infiltration and drives macrophage polarization toward the immunosuppressive M2 phenotype, thereby enhancing local immune tolerance and angiogenesis. Persistent cytokine stimulation and accumulation of inflammatory mediators contribute to a chronic low-grade inflammatory microenvironment in the thyroid. The concomitant upregulation of PD-L1 further suppresses cytotoxic T-cell activity, producing a state in which immune activation and immune inhibition coexist (54). This “inflammation–immune escape coexistence model” is now recognized as a defining immunologic feature of HPV-associated thyroid pathology. Although these canonical activities of E6 and E7 are well established, their downstream consequences are not uniform across all tissues (55–57). Most functional studies have been performed in cervical or other squamous epithelial cells, where E6/E7-driven signaling outputs vary according to cell type, nuclear receptor repertoire, and promoter context. For example, HPV18 E6 and E7 can directly interact with nuclear receptors, including the thyroid hormone receptor, and modulate their transcriptional activity in a cell-type- and promoter-dependent manner.

Molecular mimicry represents another important mechanism through which HPV may participate in thyroid autoimmunity. The viral L1 and E7 proteins share partial amino-acid sequence and structural homology with thyroid autoantigens, including thyroglobulin, thyroid peroxidase, and the TSH receptor (45, 58). Such similarity may trigger cross-reactive immune recognition, whereby antiviral responses against HPV inadvertently target thyroid tissues, thereby initiating autoimmune reactions (59). This process can result in follicular epithelial damage, lymphocytic infiltration, and fibrosis, mimicking Hashimoto-like histopathology or thyroid-associated ophthalmopathy (46). Thus, HPV-induced cross-immunity may serve as a pathogenic bridge linking viral infection to autoimmune thyroid disorders. In addition to immune inflammation, HPV infection can provoke oxidative stress. Persistent viral stimulation promotes the generation of reactive oxygen and nitrogen species (ROS and RNS), which cause DNA damage. These molecules also activate NF-κB and other inflammatory signaling pathways, creating a self-amplifying cycle between oxidative stress and inflammation (60, 61). Chronic oxidative stress exacerbates genomic instability and promotes cytokine release and immune-cell infiltration, perpetuating the inflammatory response. The mutual reinforcement between oxidative stress and inflammation leads to sustained thyroid tissue injury and structural remodeling, representing a key step in HPV-related immune dysregulation (62). As inflammation persists, HPV-infected cells gradually acquire immune-evasive and tolerogenic properties. Through secretion of IL-10 and TGF-β, these cells expand regulatory T-cell(Treg) populations and inhibit effector T-cell activation. Meanwhile, activation of the PD-L1/PD-1 signaling axis further suppresses immune clearance, creating an immunosuppressive microenvironment that favors viral persistence and modulates thyroid responsiveness to immune stimuli (63). Clinically, patients with HPV-positive tumors often show greater sensitivity to immune checkpoint inhibitors. Some individuals, however, develop immune-related thyroiditis or hypothyroidism, indicating shared signaling pathways between HPV-induced immune modulation and thyroid immune homeostasis (64). Notably, HPV and EBV have been shown to exert synergistic immunologic effects, primarily in non-thyroid cancers, although similar immune patterns have been observed in thyroid cancers as well. The two viruses cooperatively activate NF-κB and IL-6/TNF-α inflammatory pathways, suppress antiviral responses, and modulate immune-cell function through PD-L1 upregulation and viral RNA release (48, 65, 66). These interactions suggest that thyroid diseases may result from overlapping viral signals rather than a single pathogen (44). Beyond this, accumulating evidence suggests that HPV may synergize with other oncogenic viruses, such as CMV and HSV. These co-infections can further disrupt the thyroid microenvironment by amplifying chronic inflammation and immune evasion, ultimately lowering the threshold for malignant transformation (53, 66, 67). Recent studies have shown increased co-detection of HPV, EBV, and polyomaviruses in thyroid tumors, supporting the idea of a multi-virus immune dysregulation. The thyroid gland, as an immune-responsive yet partially immune-tolerant organ, is highly sensitive to viral and inflammatory stimuli (68). Under HPV infection, thyroid tissue often exhibits mild follicular epithelial hyperplasia with lymphocytic infiltration, accompanied by increased expression of IL-6, TGF-β, and PD-L1, decreased cytotoxic T-cell activity, and expansion of Tregs (69). The prolonged coexistence of inflammation and immune tolerance forms the immunological foundation of HPV-related thyroid disorders and may explain variations in immune responsiveness and thyroid dysfunction among HPV-positive individuals (70).

In addition, several immune-evasion mechanisms are distinctive to high-risk HPV rather than broadly shared across viruses. The HPV16 E5 protein retains MHC class I heavy chains within the endoplasmic reticulum and restricts their surface transport, thereby diminishing CD8^+^ T-cell recognition. Complementing this effect, E7 downregulates antigen-processing molecules such as TAP1 and LMP2, weakening peptide loading and cytotoxic activation. High-risk HPV further reinforces local immune tolerance by driving IL-10–producing dendritic cells and expanding regulatory T-cell populations. Overall, the immunological interplay between HPV and thyroid diseases represents a dynamic and evolving process. Viral infection disrupts p53 and Rb pathways, subsequently activating NF-κB and STAT3 signaling. The continuous release of proinflammatory cytokines reprograms immune cells, while the accumulation of ROS and RNS establishes a positive feedback loop that sustains inflammation (66). Molecular mimicry further triggers cross-reactive autoimmune responses, ultimately creating a microenvironment where chronic inflammation and immune tolerance coexist. Through remodeling of immune signaling, HPV acts less as a direct pathogen than as an immune modulator, influencing cell-cycle control, antigen presentation, and checkpoint regulation, and thereby reshaping thyroid immune homeostasis and inflammatory behavior (71). Taken together, these pathways outline how HPV may create a pro-inflammatory yet tolerance-oriented immune microenvironment that could influence thyroid tissue vulnerability. Future studies integrating viromics, immunomics, and thyroid microenvironmental profiling are warranted to delineate the specific molecular circuits underlying HPV-induced immune alterations in thyroid diseases. Such work will provide a theoretical foundation for targeted prevention and personalized immunotherapy in virus-related endocrine disorders (72).

### 2.4 Immunological mechanisms of HPV-induced thyroid cancer

HPV can disrupt thyroid homeostasis through immune evasion, inflammatory activation, and oxidative stress, leading to both functional dysregulation and structural damage. When viral infection persists and genomic integration occurs, sustained overexpression of the E6 and E7 oncoproteins further activates multiple cellular signaling cascades and promotes genomic instability, ultimately driving malignant transformation and tumor progression of thyroid cells. Thus, HPV-associated thyroid carcinoma can be viewed as an advanced stage of the virus-induced immunopathogenic process. Accumulating evidence indicates that HPV plays a crucial role in the initiation and development of thyroid cancer, particularly in PTC and PSCCT. The development of HPV-related thyroid cancer appears to follow a multistep process characterized by viral genome integration, cell-cycle dysregulation, activation of oncogenic signaling, inflammation-driven immune escape, and synergistic amplification with other carcinogenic factors—features typical of virus–host co-carcinogenesis. High-risk HPV types, such as 16, 18, and 33, can integrate their genomes into host DNA, disrupting the viral E2 regulatory region and causing continuous overexpression of E6 and E7 (73, 74). The E6 protein binds to the E6AP complex to promote p53 degradation, suppressing DNA repair and apoptosis, while E7 inactivates the Rb protein and releases E2F transcription factors, leading to persistent cell-cycle activation and genomic instability (75). These alterations result in abnormal cell proliferation, accumulated DNA damage, and chromosomal rearrangements, providing the molecular foundation for malignant transformation of thyroid follicular epithelial cells. E6 and E7 also interfere with BRCA1/2 signaling, impairing DNA repair capacity, accelerating mutation accumulation, and facilitating cellular dedifferentiation and malignancy. Concurrently, HPV infection activates PI3K/Akt, MAPK, NF-κB, and STAT3 pathways, which act synergistically with driver mutations such as BRAF or RAS to promote abnormal follicular cell proliferation and epithelial–mesenchymal transition (EMT) (31, 76). In some cases, HPV infection has been closely linked to squamous metaplasia of thyroid epithelium, suggesting its potential role in the dedifferentiation of differentiated thyroid carcinoma toward more aggressive phenotypes.

**Figure 1:**
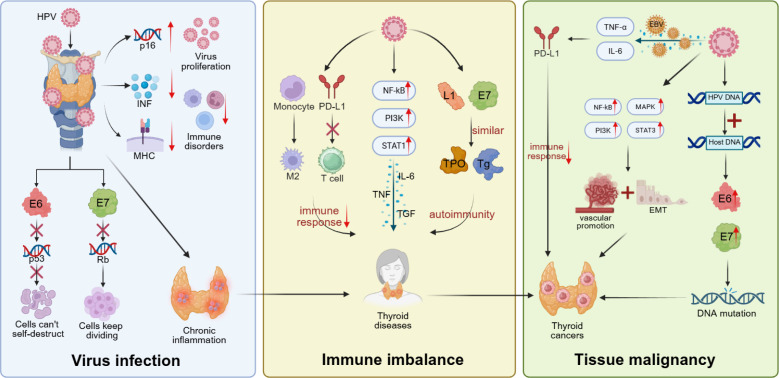
Multistep immunological and molecular pathways linking HPV infection to thyroid immune dysregulation and malignant transformation. HPV infection promotes thyroid pathology through a multistage process of immune modulation. The viral oncoproteins E6 and E7 suppress p53 and Rb pathways, dampen interferon signaling, and reduce MHC-I expression, enabling immune evasion and viral persistence. Persistent infection activates NF-κB, STAT3, and PI3K/Akt pathways, inducing cytokines such as IL-6, TNF-α, and TGF-β and creating an immunosuppressive microenvironment with M2 macrophage polarization, PD-L1 upregulation, and Treg expansion. Molecular mimicry between HPV L1/E7 and thyroid autoantigens provokes cross-reactive immunity, while excess ROS/RNS amplifies inflammation and oxidative damage. Viral DNA integration leads to sustained E6/E7 overexpression and inactivation of p53, Rb, and BRCA1/2, which, together with BRAF or RAS mutations, maintains PI3K/Akt, MAPK, and STAT3 activation. These cumulative effects foster epithelial–mesenchymal transition, angiogenesis, and immune escape, ultimately driving follicular cell dedifferentiation and thyroid carcinogenesis.

Persistent HPV infection also induces sustained activation of NF-κB and STAT3 signaling, elevating the expression of proinflammatory cytokines such as IL-6, TNF-α, and TGF-β, thereby generating a chronic inflammatory and oxidative microenvironment (77, 78). This state of inflammation promotes cell proliferation and angiogenesis while impairing immune-mediated clearance of abnormal cells, collectively providing a favorable niche for tumor development. HPV-positive thyroid cancer tissues frequently exhibit enhanced NF-κB activity and TRAF3 gene deletion, underscoring the central role of inflammation amplification in HPV-related thyroid carcinogenesis (20, 79). HPV also displays distinctive immune-escape properties. The virus replicates in a non-lytic manner within host cells, suppressing interferon signaling and antigen presentation to avoid immune recognition. Some studies suggest that HPV may reach thyroid tissue via hematogenous dissemination through peripheral blood mononuclear cells and establish latent infection in regions of immune vulnerability or chronic inflammation, where low-grade infection may persist and promote malignant transformation (66, 80). Moreover, HPV often exerts synergistic effects with other oncogenic factors. E6/E7-mediated pathway activation can act in concert with BRAF mutations or EBV infection to enhance resistance to apoptosis and anchorage-independent growth, while promoting metabolic reprogramming of tumor cells (22, 66). HPV may also interfere with thyroid hormone signaling pathways, altering cellular responsiveness to proliferative and apoptotic cues and amplifying endocrine susceptibility to neoplastic transformation. The mechanisms underlying HPV-associated thyroid carcinogenesis include viral genome integration and sustained E6/E7 overexpression, which inactivate tumor-suppressive and DNA repair pathways. These alterations cooperate with activated oncogenic signaling, chronic inflammation, and immune evasion to establish a pro-tumor microenvironment. Additional amplification through gene mutations or co-infecting viruses may further promote malignant transformation. Overall, HPV is unlikely to serve as a single etiologic agent in thyroid cancer. Rather, it functions as a co-carcinogenic immunomodulator that, through multilayered molecular events and immune microenvironmental remodeling, facilitates the initiation, progression, and dedifferentiation of thyroid malignancies(**Figure 1**).

## 3 HPV vaccination and thyroid diseases

### 3.1 Composition and immunological mechanisms of HPV vaccines

HPV vaccine is a prototypical prophylactic subunit vaccine, with its primary immunogenic component consisting of virus-like particles(VLPs) formed by the self-assembly of the major capsid protein L1, recombinantly expressed in heterologous systems. These VLPs closely mimic the structure of native viral capsids but lack viral DNA, allowing them to elicit a robust immune response without the capacity for infection or replication (81–83). Different valences of the vaccine cover distinct HPV genotypes. The bivalent vaccine(Cervarix) targets HPV16 and HPV18, the quadrivalent vaccine(Gardasil) protects against HPV6, 11, 16, and 18, and the nonavalent formulation(Gardasil 9) further extends coverage to HPV31, 33, 45, 52, and 58. The choice of adjuvant plays a critical role in shaping the immune response. Cervarix employs the AS04 adjuvant system, which enhances antigen presentation and T-cell co-stimulation, whereas the Gardasil series utilizes aluminum-based adjuvants that promote local inflammation and facilitate B-cell activation and antibody production (84–86). Following vaccination, the principal defense mechanism is humoral immunity. The repetitive L1-VLP structure effectively triggers B-cell recognition and induces high-titer neutralizing antibodies that block viral binding to the basement membrane and cell-surface receptors, thereby preventing initial viral entry into host cells (87). Nearly 100% seroconversion occurs within one month after vaccination, and antibody titers are 10–100 times higher than those induced by natural infection. Although no definitive serological threshold of protection has been established, long-term follow-up studies by the CDC and WHO have demonstrated that vaccine-induced antibodies can persist for more than a decade without significant decline (88). This durable antibody response is supported by vaccine-induced memory B cells and long-lived plasma cells, which can rapidly produce high-affinity antibodies upon antigen re-exposure, maintaining protective immunity even as circulating antibody levels wane (89, 90). In addition to humoral immunity, HPV vaccines can elicit a measurable cellular immune response. VLPs internalized by dendritic cells are presented via the MHC-II pathway to activate CD4^+^ helper T cells, which in turn promote B-cell differentiation and antibody affinity maturation. Some antigenic peptides can also undergo cross-presentation to activate CD8^+^ cytotoxic T cells, facilitating the clearance of infected epithelial cells (91).

Although HPV vaccines are primarily designed to elicit strong neutralizing antibody responses, growing evidence suggests that L1-VLP antigens exert additional immunological effects. These antigens can access the cytosolic processing pathway of dendritic cells and induce a small yet functionally important population of HPV-specific CD8^+^ T cells through cross-presentation (92, 93). After entering the cytosol via Sec61-mediated retrotranslocation, exogenous VLP-derived peptides undergo proteasomal degradation and are subsequently loaded onto MHC-I molecules in a TAP-dependent manner. This process enables efficient presentation to cytotoxic T lymphocytes, which can identify and eliminate epithelial cells expressing early viral antigens such as E6 and E7. In this way, CD8^+^ T-cell responses provide an additional layer of protection during the initial stages of infection and act in concert with neutralizing antibodies (94). Preventing persistent HPV infection through vaccination may also help limit chronic systemic immune activation. However, unlike therapeutic vaccines, prophylactic HPV vaccines are designed primarily to prevent infection rather than eliminate existing viral reservoirs. Cellular responses serve mainly to sustain immune memory, while neutralizing antibodies remain the principal determinant of protection (95, 96). Notably, the protective effects of HPV vaccination are not limited to the local mucosal or cervical environment. Emerging evidence indicates that HPV infection interacts closely with systemic immune homeostasis, and vaccine-induced immune remodeling may extend its regulatory and protective effects to other immune-sensitive organs (97). In the thyroid gland, an endocrine organ highly susceptible to immune-mediated inflammation, HPV infection is thought to promote chronic inflammation, immune dysregulation, and neoplastic transformation through persistent antigenic stimulation and immune-mediated injury. Increasing evidence suggests that HPV infection is not merely a trigger for localized epithelial disease but may also disrupt systemic immune homeostasis, contributing to various autoimmune or inflammation-associated conditions, including thyroid dysfunction and autoimmune thyroid disorders (98) (**Table 2**).

**Table 2:** Main findings of studies on HPV vaccination and thyroid diseases.

Study design	Vaccine type	Thyroid disease	Key findings	Reference
6 months–12 years follow-up from phase III trials	Bivalent, Quadrivalent and Nonavalent	immunological response to thyroid immune	Vaccines showed durable immunity and no increase in autoimmune risk	(75)
1-year follow-up after vaccination	Bivalent and Quadrivalent	HT	Risk not increased in both cohort and self-controlled analyses	(99)
6.5 years follow-up after vaccination	Bivalent and Quadrivalent	Thyroiditis	HPV vaccination not associated with increased risk of autoimmune thyroiditis	(100)
33 months follow-up after vaccination	Quadrivalent	HT and GD	Incidence of HT and GD after vaccination was below baseline population rates	(101)
Up to 2 years after vaccination	Bivalent and Quadrivalent	Autoimmune and unspecified thyroiditis	No increased risk of thyroiditis observed after HPV vaccination.	(102)
Systematic review and meta-analysis	Bivalent, Quadrivalent and Nonavalent	Autoimmune thyroid diseases	No increased risk of thyroid disease or autoimmune thyroiditis following HPV vaccination	(103)

### 3.2 Immunological mechanisms of HPV vaccination in thyroid homeostasis

Given the immunological link between HPV infection and thyroid diseases, understanding the effects of HPV vaccination provides an important extension of this relationship. Some of the immunomodulatory effects described below are supported by clinical or population-based data, whereas others remain theoretical and are extrapolated from established principles of vaccine immunology. The HPV vaccine has been widely implemented worldwide, exposing hundreds of millions of individuals to vaccine-induced immune responses. Although its anticancer efficacy has been well established, whether vaccination exerts broader effects on immune-related systems, particularly the thyroid, an organ highly sensitive to immune-mediated inflammation, remains insufficiently explored. Building on the pathogenic mechanisms of HPV infection described above, this section further examines how HPV vaccination may confer protective effects on thyroid diseases through modulation of immune homeostasis, thereby establishing a theoretical bridge between viral infection and immune protection. Multiple epidemiological and immunological studies consistently indicate that HPV vaccination is not only safe and effective in preventing virus-associated malignancies but may also exert potential protective effects on thyroid function via systemic immune stabilization. Large-scale real-world data from the United States, Europe, and East Asia have shown no significant association between HPV vaccination and autoimmune thyroid diseases, with some studies even reporting a declining trend in the incidence of hypothyroidism and thyroiditis (99, 100, 104–106). However, some studies have noted that the absence of increased risk does not necessarily imply a protective effect. A nationwide cohort study from Denmark that included more than three million individuals found no significant association between HPV vaccination and a reduced risk of autoimmune thyroiditis or hypothyroidism, indicating that vaccination does not confer measurable thyroid-specific protection (107). Evidence from large population-based cohorts provides direct support that HPV vaccination does not increase the risk of autoimmune thyroid diseases or thyroid dysfunction. These findings suggest that the benefits of HPV vaccination extend beyond antiviral defense, potentially contributing to immune tolerance maintenance and attenuation of systemic inflammation.

**Figure 2:**
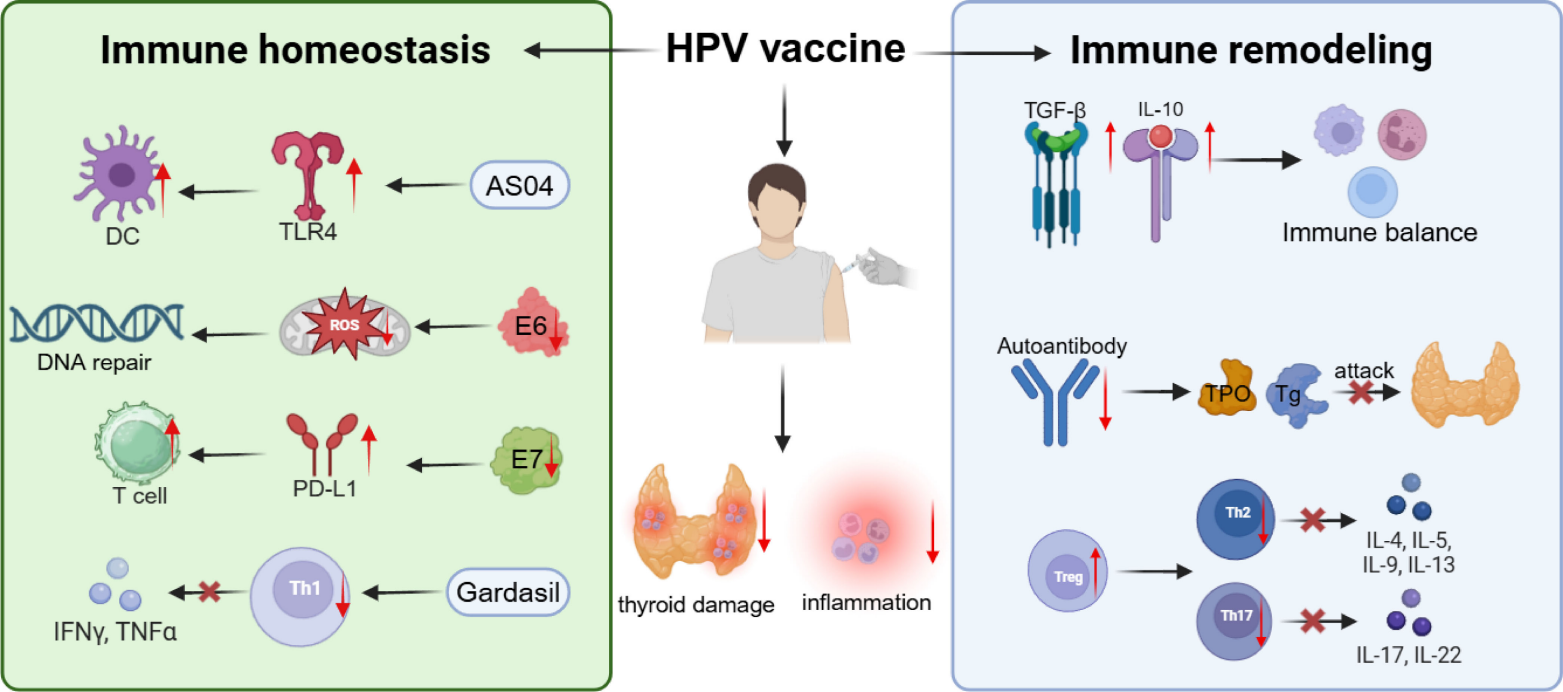
Immunomodulatory and homeostatic effects of HPV vaccination on thyroid-related immune pathways. HPV vaccination activates dendritic cells and elicits a controlled humoral response, providing antiviral protection without chronic inflammation. It increases regulatory T cells, enhances IL-10 and TGF-β secretion, and suppresses Th1/Th17 activity, shifting immunity toward a tolerance-oriented state. Adjuvants such as AS04 and aluminum salts moderately activate TLR4 and favor Th2/Treg pathways, sustaining a low-inflammatory environment. Vaccination lowers ROS levels, normalizes PD-L1 expression, and restores T-cell function, thereby preserving thyroid immune homeostasis and reducing thyroid disease risk. Vaccine-induced antibodies specifically target HPV epitopes without cross-reacting with thyroid antigens, ensuring effective antiviral defense while maintaining immune tolerance.

Linking vaccination-related observations to underlying immune responses is equally important, as it helps determine whether vaccine-induced systemic or mucosal immunity could plausibly modulate thyroid immune homeostasis. HPV vaccines may confer indirect protection against thyroid diseases through multilayered immunoregulatory processes. Rather than acting solely via antiviral mechanisms, their core effect lies in reshaping host immune homeostasis and inflammatory pathways to maintain thyroid immune tolerance. From an immunogenic standpoint, HPV vaccines are VLP-based formulations composed of L1 or L1/L2 capsid proteins but devoid of viral DNA, which elicit potent humoral responses without inducing viral replication or persistent antigenic stimulation (101). This immune response is dominated by neutralizing antibody production and dendritic cell maturation, with minimal cytotoxic activation. Consequently, it avoids nonspecific inflammatory amplification and bystander activation. Unlike natural infection, where E6/E7 proteins trigger prolonged activation of NF-κB and STAT3 pathways, vaccine-induced responses are transient and well controlled (102). This pattern of highly specific but low-inflammatory activation prevents the continuous release of proinflammatory mediators, thereby reducing systemic inflammatory burden and mitigating immune stress within the thyroid microenvironment. HPV vaccination also exhibits unique immunomodulatory properties that promote immune tolerance. Post-vaccination immune profiling has revealed increased Treg proportions and suppression of Th1 and Th17 responses, shifting the immune system toward a tolerant phenotype. These observations are supported by limited immunophenotyping studies, although further thyroid-specific evidence is still needed to establish the full extent of these effects. Elevated production of immunosuppressive cytokines such as IL-10 and TGF-β facilitates the restoration of balance between humoral and cellular immunity, preventing aberrant autoimmune reactions against self-antigens (108, 109). Moreover, vaccine-induced memory B cells are specifically directed against HPV epitopes rather than host proteins, minimizing the risk of cross-reactive autoantibody generation. This mechanism is of particular relevance to the prevention of autoimmune thyroid disorders such as HT and Graves’ disease, where autoantibody-mediated injury predominates (103). At the signaling level, HPV vaccines maintain immune homeostasis by modulating Toll-like receptor (TLR)–mediated innate responses. For instance, the AS04 adjuvant used in Cervarix activates TLR4 to induce controlled NF-κB signaling, promoting dendritic cell maturation and antigen presentation without eliciting excessive inflammation. In contrast, the aluminum adjuvant in the Gardasil series preferentially stimulates Th2 and Treg pathways, limiting hyperactive Th1-driven inflammation (110, 111). These mechanistic interpretations largely reflect established knowledge about vaccine adjuvants and their signaling profiles. Together, these adjuvant systems enable immune reprogramming by enhancing antigen-specific activation while steering the immune system toward a low-inflammatory, tolerance-prone state. Controlled immune activation also reduces oxidative stress and prevents the formation of the inflammation and immune escape feedback loop, thereby protecting the thyroid microenvironment from chronic immune-mediated injury (112). Furthermore, by preventing viral infection and persistent antigen exposure, HPV vaccination suppresses the chronic inflammation and oxidative stress that typically accompany HPV persistence. During natural infection, E6 and E7 proteins induce the accumulation of ROS and RNS, activate NF-κB signaling, and establish a self-perpetuating inflammatory loop. In contrast, vaccination eliminates viral replication and persistent antigenic stimuli, resulting in markedly lower oxidative stress levels (113, 114). A low-ROS environment helps preserve genomic stability and metabolic balance in thyroid follicular cells, reducing the likelihood of immune-mediated tissue damage (115). HPV vaccination may also indirectly modulate immune checkpoint molecule expression. Studies have shown that HPV infection, through E6/E7-mediated NF-κB activation, upregulates PD-L1 expression, leading to immune escape and T-cell exhaustion. Vaccination interrupts this pathway, restoring the normal regulation of the PD-L1/PD-1 axis (116). Downregulation of PD-L1 not only reactivates effector T-cell function but also alleviates long-term viral-induced immune tolerance within thyroid tissues. Overall, HPV vaccination exhibits a dual immunological profile characterized by selective immune activation and broad immune homeostasis maintenance (98, 117). Beyond viral neutralization, it contributes to systemic immune regulation by modulating inflammatory pathways, tolerance networks, and oxidative stress, thereby reducing the risk of immune-mediated thyroid dysfunction or structural damage (118) (**Figure 2**). A growing real-world evidence supports the long-term safety of HPV vaccination. Large population-based cohorts from France, Denmark, and the United States, with follow-up durations ranging from 5 years to over a decade, have consistently shown no increase in the incidence of autoimmune thyroid diseases or other forms of thyroid dysfunction among vaccinated individuals (101, 108, 119). Several analyses have even reported a neutral or modestly declining trend in the occurrence of hypothyroidism and thyroiditis in vaccinated populations compared with unvaccinated controls. In summary, current evidence supports robust systemic immune activation after HPV vaccination, whereas potential downstream effects on thyroid-specific immune regulation remain largely theoretical.

## 4 Conclusions and future perspectives

This study systematically integrated molecular, immunological, and epidemiological evidence to elucidate the potential roles of HPV infection and HPV vaccination in thyroid diseases, establishing a continuous research framework that links pathophysiological mechanisms with population-level validation. A comprehensive analysis of existing basic and clinical studies indicates that HPV may disrupt thyroid immune homeostasis through multiple mechanisms, including immune-inflammatory amplification, dysregulation of signaling pathways, molecular mimicry, and oxidative stress. Persistent viral infection and immune activation may foster a chronic low-grade inflammatory microenvironment in which follicular epithelial cells are continuously exposed to immune and oxidative stress, leading to cell-cycle disturbance, impaired antigen presentation, and the development of immune tolerance and escape. These processes not only contribute to thyroid functional disorders but also provide a molecular foundation for aberrant proliferation and genomic instability. Accordingly, HPV-associated thyroid carcinoma may represent an advanced stage in the virus-driven immunopathogenic continuum, reflecting a progressive transition from viral infection and immune modulation to oncogenic transformation. This concept offers new immunological insights into virus-induced endocrine tumorigenesis.

Thyroid diseases arise from the combined influence of viral, host, endocrine, and environmental factors rather than from viral activity alone (120–122). Host genetic susceptibility, thyroid-specific antigenicity, and responsiveness to systemic inflammatory cues shape how external triggers, including HPV, are integrated within the local microenvironment. Within this broader framework, HPV is best understood as a potential co-factor that interacts with pre-existing immune or endocrine vulnerabilities rather than as an isolated driver of disease. Collectively, the current evidence suggests that the relationship between HPV and thyroid diseases can be interpreted as a dynamic process evolving from viral infection and immune dysregulation to tissue remodeling and eventual tumor development. High-risk HPV types, through E6- and E7-mediated suppression of p53 and inactivation of Rb, continuously disrupt cell-cycle and antiviral signaling. They also activate immune-inflammatory pathways, including NF-κB, STAT3, and PI3K/Akt. Additionally, HPV promotes the release of cytokines such as IL-6, TNF-α, and TGF-β, creating a microenvironment characterized by coexisting chronic inflammation and immune evasion (123–125). Molecular mimicry and oxidative stress further amplify immune abnormalities. The antigenic homology of HPV L1 and E7 proteins with thyroid autoantigens may trigger cross-reactive immune responses against thyroid tissues, while virus-induced accumulation of ROS and RNS causes DNA damage and persistent signaling activation. These combined effects lead to the disruption of thyroid immune homeostasis, tissue remodeling, and functional dysregulation (126, 127). The cumulative impact of persistent infection and inflammatory stimulation drives aberrant follicular cell proliferation and genomic instability, positioning HPV-related thyroid carcinoma as an advanced outcome of the virus–immune pathogenic chain (128). This continuum illustrates the mechanistic shift of HPV from an immune regulatory factor to a cooperative oncogenic driver in thyroid pathology. In contrast, HPV vaccination may confer protective effects through the induction of highly specific humoral and cellular immune responses that block viral infection and reactivation, while restoring systemic immune balance through low-inflammatory immune remodeling (129). Vaccination enhances Treg activity, downregulates Th1 and Th17 responses, and increases the expression of immunosuppressive cytokines such as IL-10 and TGF-β, promoting a shift from an inflammatory to a tolerance-oriented immune profile (130–132). Large cohort studies conducted in different regions, including France, Denmark, and the United States, consistently show that HPV vaccination is not associated with an increased risk of thyroid dysfunction. These findings broaden the geographical and methodological basis supporting the vaccine’s safety profile. It should be noted, however, that most available studies were not specifically designed to assess thyroid-related outcomes, and this limitation should be taken into account when interpreting the current evidence. The adjuvant systems used in current vaccines support controlled antigen presentation while preventing excessive immune activation, thus maintaining equilibrium between antiviral defense and immune tolerance. This regulated immune activation lowers oxidative stress levels and corrects aberrant PD-L1/PD-1 signaling, thereby preventing virus-induced immunosuppression and protecting thyroid tissues from chronic immune injury (133).

This study presents several methodological and conceptual advances. Current molecular, immunological, and epidemiological studies provide reasonably consistent support for the major associations summarized in this review. It provides the comprehensive framework that unites HPV infection, immune regulation, vaccination, and thyroid disease within a single analytical model, connecting molecular, immunological, and epidemiological perspectives of virus–endocrine interaction. Some mechanistic interpretations remain hypothetical and should be viewed as emerging concepts rather than established causal pathways. The study also suggests that HPV-associated thyroid carcinoma may represent a later stage of virus-driven immune pathology, showing the progression from immune dysregulation to malignant transformation. This integrated perspective provides a framework for studying virus–immune interactions and immune homeostasis under multiple viral exposures. As HPV vaccination expands and research on viral co-infection advances, understanding the temporal and causal dynamics of HPV-related immune responses in thyroid diseases will be an important focus for future work. Clarifying which proposed mechanisms reflect true causal processes will require targeted validation in longitudinal and multi-omics studies. Although current studies offer suggestive links between HPV and thyroid tumors, some of the evidence comes from small cohorts or isolated case reports. Such limitations may partly explain the inconsistent detection rates across studies. In addition, some of the available studies are geographically concentrated, particularly in Asian populations, raising the possibility that differences in viral prevalence, genetic background, or environmental exposures may influence the observed associations. Such population-specific factors should be considered when assessing the generalizability of current findings, highlighting the need for larger, multi-ethnic cohorts. Integrating these molecular insights with large-scale epidemiological data may help clarify the key immune phenotypes involved in HPV-related thyroid disorders and refine risk stratification. From a public health perspective, HPV vaccination, in addition to its established efficacy against cervical and head and neck cancers, may also support thyroid immune homeostasis. Given this potential, further research is needed to explore its clinical applications. Specifically, future studies should evaluate whether HPV vaccination could be recommended for patients with thyroid diseases, particularly those with a history of viral exposure or immune dysfunction. While preliminary, prospective studies are needed to determine whether immune remodeling can provide protection against chronic immune-inflammatory thyroid disorders.
